# Impact of race on care, readmissions, and survival for patients with glioblastoma: an analysis of the National Cancer Database

**DOI:** 10.1093/noajnl/vdab040

**Published:** 2021-03-06

**Authors:** Tiffany R Hodges, Collin M Labak, Uma V Mahajan, Christina Huang Wright, James Wright, Gino Cioffi, Haley Gittleman, Eric Z Herring, Xiaofei Zhou, Kelsey Duncan, Carol Kruchko, Andrew E Sloan, Jill S Barnholtz-Sloan

**Affiliations:** 1 Department of Neurosurgery, University Hospitals Cleveland Medical Center, Case Western Reserve University, Cleveland, Ohio, USA; 2 Seidman Cancer Center and Case Comprehensive Cancer Center, Cleveland, Ohio, USA; 3 Department of Neurology, University Hospitals Cleveland Medical Center, Cleveland, Ohio, USA; 4 Department of Population and Quantitative Health Sciences, Case Western Reserve University School of Medicine, Cleveland, Ohio, USA; 5 Central Brain Tumor Registry of the United States, Hinsdale, Illinois, USA

**Keywords:** glioblastoma, health disparities, overall survival, race, readmissions

## Abstract

**Background:**

The objective of this study was to explore racial/ethnic factors that may be associated with survival in patients with glioblastoma by querying the National Cancer Database (NCDB).

**Methods:**

The NCDB was queried for patients diagnosed with glioblastoma between 2004 and 2014. Patient demographic variables included age at diagnosis, sex, race, ethnicity, Charlson–Deyo score, insurance status, and rural/urban/metropolitan location of zip code. Treatment variables included surgical treatment, extent of resection, chemotherapy, radiation therapy, type of radiation, and treatment facility type. Outcomes included 30-day readmission, 30- and 90-day mortality, and overall survival. Multivariable Cox regression analyses were performed to evaluate variables associated with race and overall survival.

**Results:**

A total of 103 652 glioblastoma patients were identified. There was a difference in the proportion of patients for whom surgery was performed, as well as the proportion receiving radiation, when stratified by race (*P* < .001). Black non-Hispanics had the highest rates of unplanned readmission (7.6%) within 30 days (odds ratio [OR]: 1.39 compared to White non-Hispanics, *P* < .001). Asian non-Hispanics had the lowest 30- (3.2%) and 90-day mortality (9.8%) when compared to other races (OR: 0.52 compared to White non-Hispanics, *P* = .031). Compared to White non-Hispanics, we found Black non-Hispanics (hazard ratio [HR]: 0.88, *P* < .001), Asian non-Hispanics (HR: 0.72, *P* < .001), and Hispanics (HR: 0.69, *P* < .001) had longer overall survival.

**Conclusions:**

Differences in treatment and outcomes exist between races. Further studies are needed to elucidate the etiology of these race-related disparities and to improve outcomes for all patients.

**Key Points**

1. Racial differences exist in mortality and readmissions for glioblastoma patients.
2. Black non-Hispanics had the highest rate of unplanned 30-day readmission (7.6%).
3. White non-Hispanics had the lowest median survival (9.03 months).

**Importance of the Study**

We conducted this study to explore racial/ethnic factors that may be associated with survival in patients with glioblastoma by querying the National Cancer Database (NCDB). We found that racial differences do exist in 30- and 90-day readmissions and mortality rates, as well as overall medial survival. This study is a useful addition to the current literature in large part due to the comprehensive scope of NCDB, as it encompasses approximately 70% of all new cancer diagnoses in the United States. Our study fills a gap in current literature surrounding race/ethnicity in glioblastoma treatment and outcomes and calls for future work to be done to further understand the reasons behind disparities in glioblastoma patient outcomes.

Glioblastoma (GBM), a World Health Organization grade IV diffuse glioma of astrocytic lineage,^1^ is the most commonly diagnosed primary malignant brain tumor with approximately 11 833 new diagnoses per year in the United States.^2^ Most recent data show that treatment with a standard of care including resection, adjuvant temozolomide-based chemotherapy, and radiation yields 5-year and 10-year survival rates of 5.4% and 2.7%, respectively.^3^ In recent years, efforts have been refocused on identifying the epidemiologic factors that contribute to the diagnosis, implemented treatment strategies, and survivorship in cancer patients, including in GBM.^2,4–11^ Past studies have shown associations between GBM risk or survival and insurance type^12–14^ or socioeconomic status.^15,16^ The datasets utilized in these studies all have limitations that analysis with the US nationwide collected dataset would otherwise yield. The National Cancer Database (NCDB) is one such central registry containing compiled data from over 1500 diverse treatment centers and represents more than 70% of newly diagnosed cancer cases nationwide.^17^ It provides a more complete representation compared to both the population-based Surveillance, Epidemiology, and End Results (SEER) database, which represents only 28% of the US population,^18^ and National Inpatient Sample, which similarly represents only 20% of hospital admissions in the United States.^19^ Although prior work on GBM has shown differences in overall survival between races, no prior study has examined different aspects of care such as readmission rates.^20^ The purpose of this study is to utilize this complete dataset to better assess the role of race/ethnicity in differences in care (eg, treatment type, readmissions) and clinical outcomes for GBM patients.

## Methods

The NCDB was queried for adults at least 18 years of age diagnosed with primary GBM between 2004 and 2014. International Classification of Diseases for Oncology, Third Edition (ICD-O-3)-Oncology codes included morphologic codes 9440, 9441, and 9442 and topographical codes C71.0–C71.9. Only patients with a histologically confirmed diagnosis of GBM were included. Baseline patient demographics, hospital characteristics, and treatment variables were compared and stratified by race/ethnicity. Patient demographic variables included age at diagnosis, sex, race (White, Black, Asian), ethnicity (Hispanic status [yes/no]), Charlson–Deyo score (identical to the Charlson Comorbidity), insurance status, and rural/urban/metropolitan location of zip code. Patients with reported race or ethnicities of “other” or “unknown” were excluded from this analysis. Hospital characteristics included treatment facility type: Community Cancer Program, Comprehensive Community Cancer Program, Academic/Research Program, and Integrated Network Cancer Program. Treatment and tumor characteristics included extent of surgical resection, unifocal or multifocal disease, type of radiation performed, and chemotherapy performed. Extent of surgical resection was categorized as no surgery, biopsy, subtotal resection, or gross total resection. Clinical outcomes examined included 30-day readmission, 30- and 90-day mortality, and overall survival. Exempt approval was obtained from the University Hospitals Institutional Review Board for this study.

### Statistical Analyses

Descriptive statistics are presented and include means and standard deviations for continuous variables and frequency and proportions for categorical variables by race/ethnicity. Chi-squared tests were applied to test differences between racial and ethnic groups. Multivariable Cox regression analysis was performed to evaluate potential variables associated with the 4 defined outcomes by race and Hispanic status (ie, race and ethnicity). Additional covariables included in the multivariable analyses were facility type, age at diagnosis, sex, primary payer (Medicare and Medicaid are government insurances in which taxpayers represent the payer), urban/rural/metro, Charlson–Deyo score, surgical resection, unifocal/multifocal, radiation, and chemotherapy. Kaplan–Meier survival curves stratified by race and ethnicity were generated. Statistical significance was set at *P* value less than .05.

## Results

Sociodemographic and treatment characteristics for the patient sample stratified by race and ethnicity (Hispanic status) are given in Table 1. A total of 103 652 patients diagnosed with GBM were identified (White non-Hispanic *n* = 90 709, Black non-Hispanic *n* = 5704, Asian *n* = 1843, and Hispanic *n* = 5396). White non-Hispanics were significantly older at the time of diagnosis when compared to other races/ethnicities. Significant differences were present in the rate of surgical resection (White non-Hispanics had the highest rate of gross total resection, 30.7%), radiation therapy (Asian non-Hispanics had the highest rate, 70.9%), and chemotherapy (White non-Hispanics had the highest rate, 65.8%).

**Table 1.** Patient Sociodemographic and Treatment Characteristics, Glioblastoma, and National Cancer Database 2004–2014

Characteristic	Overall, *N* = 93 477^a^	White Non-Hispanic, *N* = 81 900^a^	Black Non-Hispanic, *N* = 5124^a^	Asian Non-Hispanic, *N* = 1638^a^	Hispanic, *N* = 4815^a^	*P* value^b^
Facility type						<.001
Academic/Research Program	38 877 (44%)	33 806 (43%)	2422 (51%)	774 (53%)	1875 (43%)	
Community Cancer Program	5262 (5.9%)	4695 (6.0%)	218 (4.6%)	80 (5.4%)	269 (6.2%)	
Comprehensive Community Cancer Program	34 976 (39%)	31 549 (40%)	1416 (30%)	497 (34%)	1514 (35%)	
Integrated Network Cancer Program	10 161 (11%)	8723 (11%)	660 (14%)	117 (8.0%)	661 (15%)	
Unknown	4201	3127	408	170	496	
Age	64 (55–73)	64 (56–73)	60 (51–69)	61 (50–70)	60 (50–70)	<.001
Sex						.001
Female	39 748 (43%)	34 796 (42%)	2279 (44%)	715 (44%)	1958 (41%)	
Male	53 729 (57%)	47 104 (58%)	2845 (56%)	923 (56%)	2857 (59%)	
Primary payor						<.001
Medicaid	5279 (5.8%)	3486 (4.4%)	704 (14%)	218 (14%)	871 (19%)	
Medicare	40 527 (44%)	36 811 (46%)	1810 (36%)	480 (30%)	1426 (31%)	
Not insured	3433 (3.8%)	2346 (2.9%)	368 (7.4%)	133 (8.3%)	586 (13%)	
Other government	1438 (1.6%)	1245 (1.6%)	130 (2.6%)	19 (1.2%)	44 (0.9%)	
Private insurance	40 523 (44%)	36 066 (45%)	1989 (40%)	756 (47%)	1712 (37%)	
Unknown	2277	1946	123	32	176	
Urban/rural						<.001
Metro	73 567 (82%)	63 222 (81%)	4424 (89%)	1531 (98%)	4390 (95%)	
Rural	1727 (1.9%)	1656 (2.1%)	52 (1.1%)	4 (0.3%)	15 (0.3%)	
Urban	14 075 (16%)	13 354 (17%)	471 (9.5%)	31 (2.0%)	219 (4.7%)	
Unknown	4108	3668	177	72	191	
Charlson–Deyo score						<.001
0	67 094 (72%)	59 159 (72%)	3364 (66%)	1188 (73%)	3383 (70%)	
1	16 055 (17%)	13 898 (17%)	1050 (20%)	257 (16%)	850 (18%)	
2	6985 (7.5%)	6020 (7.4%)	453 (8.8%)	128 (7.8%)	384 (8.0%)	
3	3343 (3.6%)	2823 (3.4%)	257 (5.0%)	65 (4.0%)	198 (4.1%)	
Surgical resection						<.001
Biopsy	9306 (23%)	8078 (23%)	520 (22%)	183 (21%)	525 (23%)	
Gross total	12 448 (30%)	10 885 (31%)	692 (29%)	239 (28%)	632 (28%)	
None	8827 (22%)	7653 (22%)	529 (22%)	201 (23%)	444 (20%)	
Subtotal	10 297 (25%)	8790 (25%)	621 (26%)	235 (27%)	651 (29%)	
Unknown	52 599	46 494	2762	780	2563	
Focality						.11
Multifocal	7562 (19%)	6549 (19%)	403 (17%)	164 (19%)	446 (20%)	
Unifocal	32 521 (81%)	28 173 (81%)	1906 (83%)	690 (81%)	1752 (80%)	
Unknown	53 394	47 178	2815	784	2617	
Readmission						<.001
Not readmitted	85 183 (94%)	74 778 (94%)	4568 (92%)	1476 (94%)	4361 (93%)	
Readmitted	5182 (5.7%)	4376 (5.5%)	376 (7.6%)	101 (6.4%)	329 (7.0%)	
Unknown	3112	2746	180	61	125	
Radiation						<.001
Not received	27 851 (30%)	24 214 (30%)	1560 (31%)	472 (29%)	1605 (34%)	
Received	65 128 (70%)	57 266 (70%)	3534 (69%)	1152 (71%)	3176 (66%)	
Unknown	498	420	30	14	34	
Chemotherapy						<.001
Not received	31 547 (35%)	27 247 (34%)	1944 (40%)	542 (35%)	1814 (40%)	
Received	59 100 (65%)	52 338 (66%)	2960 (60%)	1027 (65%)	2775 (60%)	
Unknown	2830	2315	220	69	226	
30-day mortality						.017
Alive	65 610 (95%)	57 416 (95%)	3562 (95%)	1164 (97%)	3468 (95%)	
Dead	3587 (5.2%)	3163 (5.2%)	204 (5.4%)	39 (3.2%)	181 (5.0%)	
Unknown	24 280	21 321	1358	435	1166	
90-day mortality						<.001
Alive	58 095 (84%)	50 716 (84%)	3204 (86%)	1070 (90%)	3105 (86%)	
Dead	10 859 (16%)	9694 (16%)	540 (14%)	121 (10%)	504 (14%)	
Unknown	24 523	21 490	1380	447	1206	
Survival months	9 (3–18)	9 (3–18)	10 (4–20)	12 (4–24)	10 (4–21)	<.001
Unknown	4	3	0	0	1	
Vital status						<.001
Alive	9985 (11%)	7836 (9.6%)	751 (15%)	331 (20%)	1067 (22%)	
Dead	83 492 (89%)	74 064 (90%)	4373 (85%)	1307 (80%)	3748 (78%)	

^a^Statistics presented: *n* (%); median (IQR).

^b^Statistical tests performed: chi-square test of independence; Kruskal–Wallis test.

Multivariable logistic regression models for 30-day readmission by race/ethnicity are given in Table 2. Black non-Hispanics had the highest rates of unplanned readmission within 30 days, as a significant difference was seen comparing Black non-Hispanics to White non-Hispanics on multivariable modeling (odds ratio [OR] 1.39, 95% confidence interval [CI] 1.15–1.6, *P* < .001). Significant associations were also seen with facility type, Charlson–Deyo score, surgical resection, and focality.

**Table 2.** Multivariable Logistic Regression Model for 30-Day Readmission, Glioblastoma, and National Cancer Database 2004–2014

Characteristic	Event *N*	OR	95% CI	*P* value
Race ethnicity				
White non-Hispanic	1589	—	—	
Black non-Hispanic	139	1.39	1.15–1.66	<.001
Asian non-Hispanic	43	1.24	0.89–1.68	.2
Hispanic	107	1.11	0.89–1.35	.3
Facility type				
Academic/Research Program	825	—	—	
Community Cancer Program	77	0.89	0.70–1.13	.4
Comprehensive Community Cancer Program	687	1.02	0.92–1.13	.7
Integrated Network Cancer Program	289	1.34	1.16–1.54	<.001
Age		1.00	0.99–1.01	.9
Sex				
Female	777	—	—	
Male	1101	1.02	0.92–1.12	.7
Primary payer				
Medicaid	119	—	—	
Medicare	874	0.96	0.77–1.20	.7
Not insured	81	1.11	0.83–1.49	.5
Other government	36	1.03	0.69–1.51	.9
Private insurance	768	0.89	0.73–1.09	.3
Urban/rural				
Metro	1589	—	—	
Rural	38	1.12	0.79–1.55	.5
Urban	251	0.90	0.78–1.03	.12
Charlson–Deyo score				
0	1163	—	—	
1	383	1.23	1.09–1.39	<.001
2	227	1.76	1.51–2.04	<.001
3	105	1.56	1.26–1.92	<.001
Surgical resection				
Biopsy	467	—	—	
Gross total	698	1.16	1.03–1.32	.015
None	74	0.15	0.11–0.19	<.001
Subtotal	639	1.25	1.11–1.42	<.001
Focality				
Multifocal	391	—	—	
Unifocal	1487	0.77	0.68–0.86	<.001
Radiation				
Not received	484	—	—	
Received	1394	0.97	0.82–1.15	.7
Chemotherapy				
Not received	553	—	—	
Received	1325	0.96	0.81–1.12	.6

OR, odds ratio; CI, confidence interval.

Results of multivariable logistic regression models for 30-day and 90-day mortality by race/ethnicity are given in Tables 3 and 4, respectively. Asian non-Hispanics had the lowest odds of 30-day and 90-day mortality when compared to other races. White non-Hispanics had the highest odds of 30-day and 90-day mortality (although Black non-Hispanics had the highest unadjusted 30-day mortality rate, Table 1). A significant difference in 30-day mortality was seen on multivariable analysis when comparing Asian non-Hispanics and White non-Hispanics (OR 0.52, 95% CI 0.28–0.91, *P* = .031). Significant differences were seen in 90-day mortality between White non-Hispanics and Asian non-Hispanics (OR 0.64, 95% CI 0.46–0.89, *P* = .009) and Hispanics (OR 0.648, 95% CI 0.528–0.794, *P* < .001), though not Black non-Hispanics (OR 0.88, 95% CI 0.73–1.06, *P* = .2).

**Table 3.** Multivariable Logistic Regression Model for 30-Day Mortality, Glioblastoma, and National Cancer Database 2004–2014

Characteristic	Event *N*	OR	95% CI	*P* value
Race ethnicity				
White non-Hispanic	1019	—	—	
Black non-Hispanic	64	0.88	0.66–1.17	.4
Asian non-Hispanic	13	0.52	0.28–0.91	.031
Hispanic	58	0.84	0.61–1.13	.3
Facility type				
Academic/Research Program	455	—	—	
Community Cancer Program	53	1.00	0.72–1.37	>.9
Comprehensive Community Cancer Program	483	1.10	0.95–1.27	.2
Integrated Network Cancer Program	163	1.23	1.00–1.51	.049
Age		1.02	1.01–1.02	<.001
Sex				
Female	447	—	—	
Male	707	1.27	1.11–1.45	<.001
Primary payer				
Medicaid	56	—	—	
Medicare	718	1.09	0.79–1.53	.6
Not insured	46	1.20	0.77–1.86	.4
Other government	16	0.76	0.40–1.39	.4
Private insurance	318	1.08	0.79–1.50	.6
Urban/rural				
Metro	946	—	—	
Rural	21	0.87	0.52–1.39	.6
Urban	187	0.93	0.77–1.11	.4
Charlson–Deyo score				
0	586	—	—	
1	310	1.62	1.38–1.91	<.001
2	142	1.50	1.21–1.85	<.001
3	116	2.33	1.82–2.96	<.001
Surgical resection				
Biopsy	417	—	—	
Gross total	327	0.67	0.57–0.80	<.001
None	31	1.61	1.01–2.51	.039
Subtotal	379	0.86	0.73–1.01	.073
Focality				
Multifocal	233	—	—	
Unifocal	921	0.73	0.62–0.87	<.001
Radiation				
Not received	1095	—	—	
Received	59	0.04	0.03–0.06	<.001
Chemotherapy				
Not received	1074	—	—	
Received	80	0.21	0.15–0.27	<.001

OR, odds ratio; CI, confidence interval.

**Table 4.** Multivariable Logistic Regression Model for 90-Day Mortality, Glioblastoma, and National Cancer Database 2004–2014

Characteristic	Event *N*	OR	95% CI	*P* value
Race ethnicity				
White non-Hispanic	3282	—	—	
Black non-Hispanic	193	0.88	0.73–1.06	.2
Asian non-Hispanic	54	0.64	0.46–0.89	.009
Hispanic	156	0.65	0.53–0.79	<.001
Facility type				
Academic/Research Program	1450	—	—	
Community Cancer Program	187	1.20	0.99–1.46	.065
Comprehensive Community Cancer Program	1594	1.32	1.20–1.45	<.001
Integrated Network Cancer Program	454	1.18	1.03–1.35	.017
Age		1.04	1.03–1.05	<.001
Sex				
Female	1561	—	—	
Male	2124	1.05	0.97–1.15	.2
Primary payer				
Medicaid	180	—	—	
Medicare	2369	0.93	0.76–1.14	.5
Not insured	134	1.11	0.84–1.46	0.5
Other government	55	0.69	0.47–1.00	.056
Private insurance	947	0.75	0.61–0.91	.004
Urban/rural				
Metro	3005	—	—	
Rural	64	0.75	0.54–1.02	.074
Urban	616	1.02	0.91–1.15	.7
Charlson–Deyo score				
0	2063	—	—	
1	881	1.44	1.29–1.59	<.001
2	451	1.70	1.48–1.95	<.001
3	290	2.14	1.79–2.55	<.001
Surgical resection				
Biopsy	1250	—	—	
Gross total	1045	0.57	0.51–0.63	<.001
None	75	1.22	0.88–1.69	.2
Subtotal	1315	0.92	0.83–1.02	.10
Focality				
Multifocal	782	—	—	
Unifocal	2903	0.60	0.54–0.67	<.001
Radiation				
Not received	2508	—	—	
Received	1177	0.22	0.20–0.25	<.001
Chemotherapy				
Not received	2654	—	—	
Received	1031	0.27	0.24–0.31	<.001

OR, odds ratio; CI, confidence interval.

**Figure 1.**
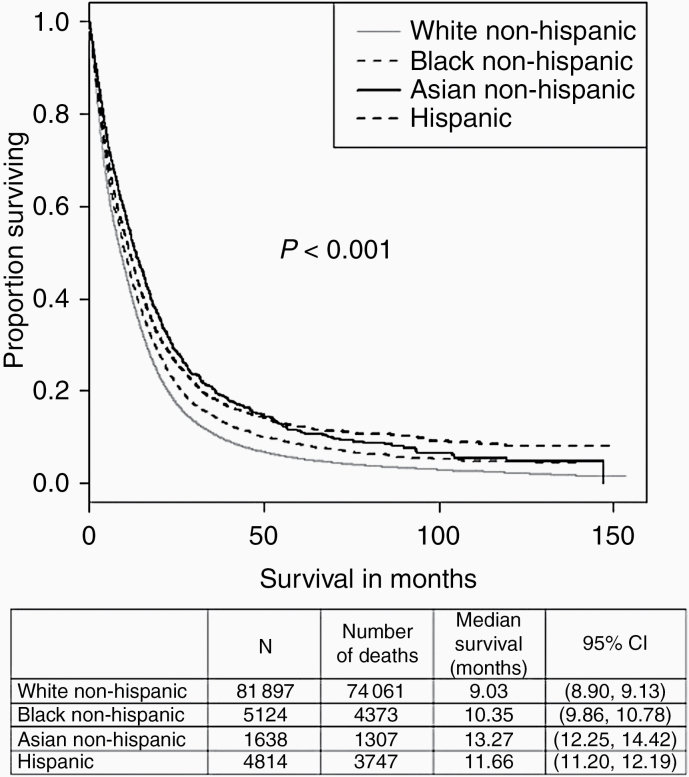
Kaplan–Meier survival analysis by race and ethnicity, glioblastoma, and National Cancer Database 2004–2014. *Median survival with 95% CI also shown by race/ethnicity.

Results of multivariable Cox Proportional Hazards modeling of race/ethnicity for overall survival are given in Table 5. When compared to a reference group of White non-Hispanics, Black non-Hispanics (hazard ratio [HR]: 0.88, 95% CI 0.83–0.92, *P* < .001), Asian non-Hispanics (HR: 0.72, 95% CI 0.65–0.73, *P* < .001), and Hispanics (HR: 0.69, 95% CI 0.65–0.73, *P* < .001) all had significantly lower overall survival HRs. Results of multivariable Cox Proportional Hazards modeling excluding from analysis individuals who died within 90 days are given in Supplementary Table 1. White non-Hispanics still had a significantly lower overall survival (ratios were as follows: Black non-Hispanics [HR: 0.93, 95% CI 0.87–0.99, *P* = .030], Asian non-Hispanics [HR: 0.70, 95% CI 0.36–0.79, *P* < .001], and Hispanics [HR: 0.71, 95% CI 0.66–0.77, *P* < .001]). Kaplan–Meier survival curves by race and median survival are demonstrated in Figure 1. Race was significantly associated with adjusted overall survival (*P* < .001), with White non-Hispanic having the lowest median survival (9.03 months) and Asian non-Hispanic having the highest (13.27 months). Kaplan–Meier survival curves by race and median survival, excluding from analysis individuals who died within 90 days, are demonstrated in Supplementary Figure 1. Race was still significantly associated with adjusted overall survival (*P* < .001), with White non-Hispanic having the lowest median survival (14.2 months) and Asian non-Hispanic having the highest (18.2 months).

**Table 5.** Multivariable Cox Proportional Hazards Model for Overall Survival, Glioblastoma, and National Cancer Database 2004–2014

Characteristic	Event *N*	HR	95% CI	*P* value
Race ethnicity				
White non-Hispanic	25 525	—	—	
Black non-Hispanic	1495	0.88	0.83–0.92	<.001
Asian non-Hispanic	507	0.72	0.65–0.78	<.001
Hispanic	1224	0.69	0.65–0.73	<.001
Facility type				
Academic/Research Program	12 272	—	—	
Community Cancer Program	1517	1.11	1.05–1.17	<.001
Comprehensive Community Cancer Program	11 455	1.14	1.12–1.17	<.001
Integrated Network Cancer Program	3507	1.19	1.14–1.23	<.001
Age		1.03	1.02–1.03	<.001
Sex				
Female	12 105	—	—	
Male	16 646	1.07	1.05–1.10	<.001
Primary payer				
Medicaid	1500	—	—	
Medicare	14 265	0.96	0.91–1.02	.2
Not insured	951	0.96	0.88–1.04	.3
Other government	477	0.94	0.85–1.04	.2
Private insurance	11 558	0.85	0.80–0.89	<.001
Urban/rural				
Metro	23 767	—	—	
Rural	549	1.06	0.97–1.15	.2
Urban	4435	1.07	1.04–1.11	<.001
Charlson–Deyo score				
0	19 579	—	—	
1	5537	1.20	1.17–1.24	<.001
2	2368	1.25	1.19–1.30	<.001
3	1267	1.43	1.35–1.51	<.001
Surgical resection				
Biopsy	6623	—	—	
Gross total	8317	0.76	0.74–0.79	<.001
None	6455	1.48	1.43–1.53	<.001
Subtotal	7356	0.98	0.95–1.02	.3
Focality				
Multifocal	5756	—	—	
Unifocal	22 995	0.75	0.73–0.77	<.001
Radiation				
Not received	8197	—	—	
Received	20 554	0.69	0.67–0.72	<.001
Chemotherapy				
Not received	9502	—	—	
Received	19 249	0.60	0.58–0.62	<.001

HR, hazard ratio; CI, confidence interval.

## Discussion

The purpose of this study was to examine the NCDB for an association between race and overall survival, 30-day and 90-day mortality for patients with GBM. Though the NCDB has its own limitations with regard to case coverage and reporting, its scope is quite comprehensive, covering approximately 70% of all new cancer diagnoses in the United States according to a recent comparison of national databases.^17^

Our study demonstrates that survival for GBM patients is in part associated with race. Such an association has also been found in various other cancers. Multiple NCDB analyses reporting on other cancer types note race as a significant factor in survival, notably in breast cancer,^21^ T-cell lymphoma,^22^ uterine cancer,^23^ and endometrial cancer.^24^ Moreover, previous analyses on GBM have similarly demonstrated that non-Hispanic Whites have the poorest survival, even account for differences in treatment. A SEER database GBM study found that Hispanics have the highest survival, followed by Blacks, and then Asian/Pacific Islanders, relative to Whites.^20^ White patients were also shown to have the poorest survival in other GBM datasets, including the Central Brain Tumor Registry of the United States.^2^ However, these studies do not address variation in short-term survival (eg, 30-day and 90-day mortality), nor do they address issues related to quality of care as reflected by 30-day readmissions.

We hypothesized if considerations having to do with access to care might vary by race. Our analysis demonstrates that non-Hispanic Whites are more likely than Hispanics and non-Hispanic Blacks to receive standard of care treatment. Specifically, treatment use is significantly lower in 2–3 race/ethnicity groups compared to White non-Hispanics, including surgical resection and each adjuvant therapy (radiation therapy or chemotherapy). There are several reports that have observed the survival benefits of undergoing treatment in high-volume academic centers compared to lower-volume community centers.^25,26^ Similar findings have been reported for patients undergoing isolated radiation therapy.^27^ There has been recently published data on facility volume and outcomes specifically in GBM patients, demonstrating a substantial decrease in prolonged length of stay, readmission rates, and mortality in patients who sought treatment at academic and/or high-volume centers.^28^ In fact, multidisciplinary “Tumor Board” conferences have shown to lead to better outcomes, including a higher likelihood of patients receiving adjuvant treatment.^29^ With regard to the selection of treatment facility, our analysis shows that a majority of GBM patients seek treatment at either a Comprehensive Community Cancer Program or Academic/Research Program irrespective of race, which represents high-volume facility types. This is in line with past analysis demonstrating GBM patients who travel farther to receive care at high-volume centers have superior postoperative outcomes compared to patients who receive care locally at low-volume centers.^30^ This may reflect the more comprehensive diagnostic capabilities of such centers, as well as the presence of oncologic care pathways that provide continuous and multidisciplinary care from the time of diagnosis through resection and adjuvant therapy. This may also reflect limitations in the ability of community programs to access services such as advanced MRI imaging or radiation therapy. Such access has previously been demonstrated as a factor in survival in other cancer types.^31^

Our analysis found that differences in 30-day readmissions were present; non-Hispanic Whites are less likely than Hispanics and non-Hispanic Blacks to be re-admitted, suggesting disparities in short-term outcomes. This may reflect the existence of higher rates of perioperative complications not captured by the NCDB. Racial disparities in short-term perioperative outcomes have been widely reported for other oncologic procedures,^32–36^ particularly when comparing Black to Caucasian populations. Additionally, readmissions may be correlated to treatment differences by race, specifically, in regard to pain management. Both oncologic and non-oncologic evidence suggest that Blacks, when compared to other races, have both higher pain scores and higher rates of inadequately treated pain.^37,38^ While the NCDB does not have sufficient granularity in their data to tease out these specific reasons for readmission, multiple hypotheses that have been proven in the literature include higher rates of perioperative complications, inadequately managed periprocedural pain control, racial bias in discharge planning, differences in communication of discharge expectations, and inadequate provision of socioeconomic support services.^39–41^ Additionally, a growing body of evidence suggests that racism, ie, systemic bias, rather than race itself may be the driving factor behind observed healthcare disparities.^42,43^ Cumulatively these reasons may be contributing factors to the higher rates of 30-day readmission and mortality rates for the Black non-Hispanic population.

Despite the higher rates of treatment and lower readmissions, White non-Hispanics exhibited the lowest overall survival, demonstrated by our multivariable cox proportional hazards model. Although this may be partially attributable to older ages at diagnosis in White non-Hispanics, our multivariable model did adjust for age. We found that survival was highest in Asians, followed by Hispanics and Blacks relative to Caucasians. Importantly, this finding was durable even after excluding from analysis the patient who died within the first 90 days, that is, Whites continue doing worse even beyond the 90-day window, suggesting biology may underlie these differences.

This study does have a number of limitations, as database studies are inherently limited by selection bias, missing data, and confounding factors. The overwhelming majority of GBM patients in the NCDB are White non-Hispanic. Other similar such studies utilizing the NCDB have samples that are up to 90.5% White patients.^12^ However, this in part reflects the known higher incidence in White patients. The Central Brain Tumor Registry of the US’s 2011–2015 primary brain tumor report shows that the incidence rate of GBM was higher in White patients (3.47) compared to Black patients (1.80) and Asian patients (1.57).^2^ While the NCDB captures data for approximately 70% of all new cancer diagnoses, only hospitals with Commission on Cancer accreditation are included, which only applies to about 30% of 5000 hospitals in the United States. This may present a selection bias for the study population, as racial differences may be present in the use of high-volume Commission on Cancer accredited hospitals.^44^ Furthermore, only patients with a histologically confirmed diagnosis were included in this dataset, which may limit inclusion of the elderly, those with a late diagnosis, and disadvantaged individuals without access to a histologically confirmed diagnosis. This could also be an indirect contributor to racial differences in survival. Exclusion of patients with undiagnosed cancers or death prior to diagnosis may also influence findings. Additionally, analyzing mortality as an outcome for a disease with as rapid a course as GBM presents the potential confounder of time of presentation influencing the duration of postoperative survival measured. With regard to missing data, most patient data did not have Karnofsky Performance Status, MGMT methylation status, extent of resection, extent of adjuvant therapy, despite all having an important relationship with survival data^45,46^; therefore, these had to be excluded in the multivariable analyses. As with virtually all multi-institutional registries, miscoding or erroneous entry of data variables must be acknowledged. Additional important predictors such as molecular markers were not present in this dataset, which can have significant impacts on survival as well as may have racial associations which should be investigated in future studies. Finally, race is an elusive concept, sometimes imposed by external sources (the racial NCDB may not reflect all races) or those internally defined. Healthcare outcomes can differ across communities and encompass socioeconomic factors as well as cultural and access issues that are built into the racialism that creates some and fractures many other communities.^47^

## Conclusions

Ultimately, our findings reiterate the well-known disparities that exist in healthcare as a function of race and should alert care providers and researchers to the continued need for improvement in studying and addressing these issues. Given that GBM is the most common primary brain tumor, it is likely that such findings related to racial/ethnic differences may be true in other primary brain tumors, as our group has demonstrated similar results in gliosarcoma.^48^ We call for further work to be done to understand the sources behind the observed disparities in GBM patient treatment, readmissions, and outcomes, so these disparities can ultimately be overcome. Future studies should be focused on addressing such disparities, and such national databases can continue to contribute to this work in the manner in which they collect patient data.

## Supplementary Material

vdab040_suppl_Supplementary_Figure_1

vdab040_suppl_Supplementary_Table_1

## Notes

**Authorship Statement** Conception: T.H., A.S., and J.B.S.; data collection: T.H., C.H.W., J.W., and H.G.; statistical analysis: G.C., H.G., and C.K.; drafting—original: C.M.L. and U.V.M.; drafting—revisions and editing: C.M.L., U.V.M., E.H., X.Z., and K.D.; approved final version: all authors; study supervision: T.H., A.S., and J.B.S.
