# Supplementary figures and images for: Impact of race on care, readmissions, and survival for patients with glioblastoma: an analysis of the National Cancer Database
Source: Neurooncol Adv. 2021 Mar 6;3(1):vdab040. doi: 10.1093/noajnl/vdab040 (PMC8086235; doi:10.1093/noajnl/vdab040)

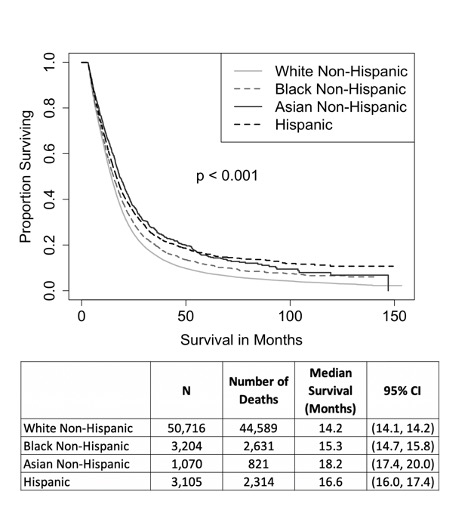

Supplement: vdab040_suppl_Supplementary_Figure_1 [file vdab040_suppl_supplementary_figure_1.png]
