# Supplementary material for: Impact of race on care, readmissions, and survival for patients with glioblastoma: an analysis of the National Cancer Database
Source: Neurooncol Adv. 2021 Mar 6;3(1):vdab040. doi: 10.1093/noajnl/vdab040 (PMC8086235; doi:10.1093/noajnl/vdab040)
Supplement: vdab040_suppl_Supplementary_Table_1 [file vdab040_suppl_supplementary_table_1.docx]

**Supplementary Table 1. Multivariable Cox Proportional Hazards Model for Overall Survival excluding patients that died within 90 days, Glioblastoma, National Cancer Database (NCDB) 2004-2014.**

| Characteristic | Event N | HR^1^ | 95% CI^1^ | p-value |
| --- | --- | --- | --- | --- |
| **Race Ethnicity** |  |  |  |  |
| White Non-Hispanic | 15,611 | — | — |  |
| Black Non-Hispanic | 897 | 0.93 | 0.87, 0.99 | 0.030 |
| Asian Non-Hispanic | 305 | 0.70 | 0.63, 0.79 | <0.001 |
| Hispanic | 756 | 0.71 | 0.66, 0.77 | <0.001 |
| **Facility Type** |  |  |  |  |
| Academic/Research Program | 8,109 | — | — |  |
| Community Cancer Program | 815 | 1.15 | 1.07, 1.23 | <0.001 |
| Comprehensive Community Cancer Program | 6,508 | 1.14 | 1.10, 1.18 | <0.001 |
| Integrated Network Cancer Program | 2,137 | 1.17 | 1.12, 1.23 | <0.001 |
| **Age** |  | 1.03 | 1.02, 1.03 | <0.001 |
| **Sex** |  |  |  |  |
| Female | 7,138 | — | — |  |
| Male | 10,431 | 1.08 | 1.05, 1.12 | <0.001 |
| **Primary Payer** |  |  |  |  |
| Medicaid | 954 | — | — |  |
| Medicare | 7,375 | 0.93 | 0.86, 1.00 | 0.059 |
| Not Insured | 586 | 0.91 | 0.82, 1.01 | 0.082 |
| Other Government | 324 | 0.96 | 0.85, 1.09 | 0.6 |
| Private Insurance | 8,330 | 0.85 | 0.80, 0.91 | <0.001 |
| **Urban Rural** |  |  |  |  |
| Metro | 14,620 | — | — |  |
| Rural | 326 | 1.11 | 1.00, 1.24 | 0.057 |
| Urban | 2,623 | 1.09 | 1.04, 1.13 | <0.001 |
| **Charlson Deyo Score** |  |  |  |  |
| 0 | 12,631 | — | — |  |
| 1 | 3,114 | 1.15 | 1.11, 1.20 | <0.001 |
| 2 | 1,232 | 1.14 | 1.08, 1.21 | <0.001 |
| 3 | 592 | 1.36 | 1.25, 1.48 | <0.001 |
| **Surgical Resection** |  |  |  |  |
| Biopsy | 4,174 | — | — |  |
| Gross Total | 7,218 | 0.83 | 0.80, 0.86 | <0.001 |
| None | 202 | 1.04 | 0.90, 1.20 | 0.6 |
| Subtotal | 5,975 | 1.05 | 1.01, 1.09 | 0.018 |
| **Focality** |  |  |  |  |
| Multifocal | 2,751 | — | — |  |
| Unifocal | 14,818 | 0.72 | 0.69, 0.75 | <0.001 |
| **Radiation** |  |  |  |  |
| Not Received | 2,369 | — | — |  |
| Received | 15,200 | 1.03 | 0.97, 1.10 | 0.3 |
| **Chemotherapy** |  |  |  |  |
| Not Received | 2,940 | — | — |  |
| Received | 14,629 | 0.69 | 0.65, 0.73 | <0.001 |
| ^1^HR = Hazard Ratio, CI = Confidence Interval | | | | |
